# A New Family of Electron-Bifurcating Enzymes Represented by Ferredoxin- and NAD-Dependent Glycerol-3-Phosphate Oxidoreductase

**DOI:** 10.1021/acs.biochem.6c00512

**Published:** 2026-10-07

**Authors:** Gerrit J. Schut, Farris L. Poole, Michael W. W. Adams

**Affiliations:** Department of Biochemistry & Molecular Biology, 1355 University of Georgia, Athens, Georgia 30602, United States

## Abstract

Glycerol is abundant in natural environments and is utilized by many microbes. In aerobes, it is typically converted, via glycerol-3-phosphate (G3P), to dihydroxyacetone phosphate (DHAP) by a quinone-linked dehydrogenase thereby coupling glycolysis and respiration. Under anaerobic conditions, an NAD^+^-dependent dehydrogenase has been proposed to oxidize G3P to DHAP in several bacteria but this reaction is thermodynamically unfavorable. Herein, we have purified a heterotrimeric electron bifurcating enzyme from the archaeon *Pyrococcus furiosus* where the simultaneous oxidation of both G3P (E_o_′ −190 mV) and reduced ferredoxin (E_m_ ∼ −450 mV) enables reduction of NAD^+^(E_o_′ −320 mV) and production of DHAP. Surprisingly, this ferredoxin-dependent NAD^+^ G3P oxidoreductase (FngABC) is unrelated evolutionarily to the four known families of bifurcating enzyme. Analysis by UV–visible spectroscopy and of its predicted structure suggest FngABC has a nonclassical bifurcation mechanism. Phylogenetic and genome context analyses of 4,273 *P. furiosus* FngA homologues revealed they are widespread with ∼35% found mainly in Clostridia as FngABC and ∼10% found mainly in Actinomyces as FngAB, most of which utilize polyols in addition to glycerol. The remaining ∼40% are present in Gammaproteobacteria and halophilic archaea but here FngA is associated with homologues of *E. coli* GlpBC, which are unrelated to FngBC, and these enzymes typically only use glycerol. It is therefore proposed that *P. furiosus* FngABC and the previously characterized *E. coli* GlpABC are disparate members of a new electron bifurcating family widespread in the microbial world that are involved in the metabolism of a range of sugars as well as glycerol.

## Graphical abstract

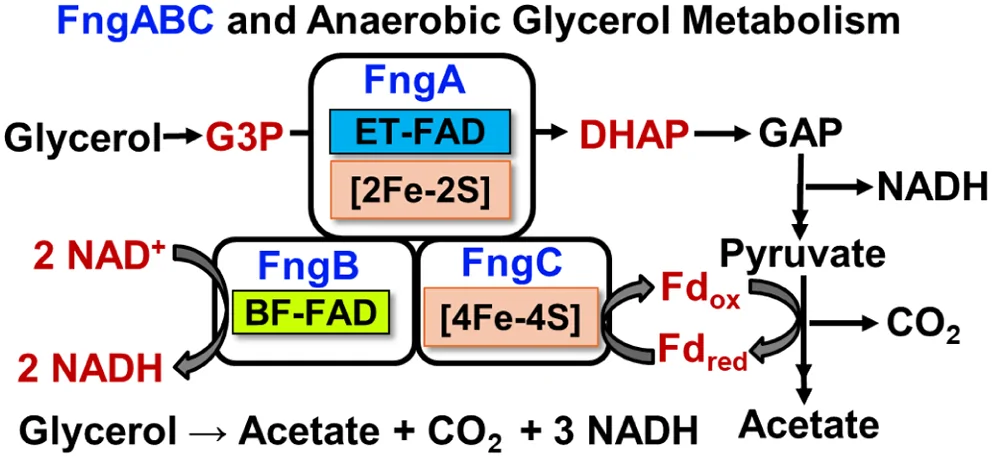

## Introduction

Glycerol (1,2,3-propanetriol) is a simple compound that is widespread in natural environments and functions as both a structural molecule and as sources of carbon and energy. It serves as the backbone of membrane phospholipids and storage triglycerides across all domains of life.^1^ Glycerol and its derivatives can also act as compatible solutes, particularly in hypersaline environments.^2^ Glycerol supports the growth of a wide range of organisms and is metabolized through multiple routes depending on the presence of external electron acceptors.^3^ Under aerobic conditions, glycerol oxidation is coupled to the quinone pool and aerobic respiration, whereas under anaerobic conditions it can still rely on the quinone pool if other electron acceptors can be used. Glycerol catabolism proceeds primarily through two distinct biochemical pathways, both of which converge on dihydroxyacetone phosphate (DHAP), a glycolytic intermediate in central carbon metabolism. In the glycerol-3-phosphate (G3P) pathway, glycerol is phosphorylated using ATP upon entry into the cell by glycerol kinase (GlpK)^4^ and G3P is subsequently oxidized to DHAP by a G3P dehydrogenase-type enzyme.^3^ In the alternative dihydroxyacetone (DHA) pathway, glycerol is first oxidized to DHA by glycerol dehydrogenase, after which DHA is converted to DHAP by an ATP-dependent DHA kinase.^5,6^

Because glycerol is a highly reduced substrate, its anaerobic metabolism under nonrespiratory conditions imposes substantial redox constraints. The DHAP and DHA degradation pathways generate NADH and this must be reoxidized to NAD^+^ to sustain metabolic flux. This can be achieved by a variety of “electron sink” pathways, diverting excess electrons into reduced end products such as 1,3-propanediol, alcohols or butyrate, or alternatively, through the production of hydrogen gas.^7^ Among these routes, the biological production of 1,3-propanediol from glycerol represents a high-value microbial process, as 1,3-propanediol is a versatile platform chemical with broad industrial applications.^8,9^ It is produced via a branched metabolic pathway^10^ where one branch oxidizes glycerol to more oxidized metabolites, such as acetate and generates reducing equivalents, while the parallel branch consumes these electrons to reduce glycerol-derived intermediates to 1,3-propanediol, thereby maintaining intracellular redox balance.

*E. coli* is one of the best-studied organisms that grow on glycerol. It possesses both the DHA and DHAP pathways, which are preferentially utilized under fermentative and respiratory conditions, respectively.^11^ Depending on the physiological conditions, *E. coli* can maintain redox balance through the production of ethanol, mixed acid fermentation products, molecular hydrogen or via aerobic or anaerobic respiration.^7,11,12^ Under aerobic conditions, glycerol is oxidized through the G3P pathway to DHAP using the quinone-linked G3P dehydrogenase (GlpD), which feeds electrons into the respiratory chain.^7^ During anaerobic growth with alternative electron acceptors such as fumarate or nitrate, *E. coli* instead employs a membrane-associated, trimeric G3P dehydrogenase (GlpABC^12^). Notably, GlpABC is also widely conserved in haloarchaea, where it is essential for glycerol utilization under respiratory conditions.^13^

A fundamental problem in glycerol metabolism by nonrespiratory anaerobes is that the glycerol/DHA (E_o_′ −190 mV) and G3P/DHAP (E_o_′ −190 mV) redox couples are more positive than that of NADH/NAD^+^ (E_o_′ −320 mV) thereby prohibiting the efficient coupling of glycerol or G3P oxidation to the reduction of NAD^+^. Nevertheless, in anaerobic acetogens such as *Acetobacterium woodii*, the oxidation of glycerol was proposed to proceed through a NAD^+^-linked G3P dehydrogenase where excess reducing equivalents were consumed by the reductive conversion of CO_2_ to acetate by the Wood-Ljungdahl pathway, even though glycerol-grown cells had extremely low G3P dehydrogenase activity using NAD^+^ as the electron acceptor.^14,15^ The fermentative bacterium *Thermotoga maritima* also grows on glycerol to produce acetate, H_2_, and CO_2_ as end products.^16^ It was proposed that *T. maritima* overcomes the thermodynamic problem of glycerol oxidation by employing reverse electron transport through its Rnf complex to convert excess NADH into reduced ferredoxin (Fd), thereby utilizing a ion gradient to dispose of reducing equivalents as H_2_ via a bifurcating hydrogenase.^16^ However, such a mechanism does not address the redox imbalance between G3P and NAD^+^ and although *T. maritima* contains a homologue of the *A. woodii* NAD^+^-dependent G3P dehydrogenase, its activity was not measured.^16^

The thermodynamic limitation of coupling glycerol oxidation to NAD^+^ reduction under anaerobic nonrespiratory conditions is analogous to the situation of lactate oxidation to pyruvate (E_o_′ −190 mV). In *A. woodii*, this is overcome by the presence of an electron bifurcating lactate dehydrogenase (LCT). This simultaneously couples the thermodynamically unfavorable coupling of lactate oxidation to NAD^+^ reduction to the energetically favorable reduction of NAD^+^ using reduced Fd (Fd_red_, E′ ∼ −500 mV) as the electron donor.^17^ It was subsequently shown that the human gut acetogen, *Eubacterium limosum*, uses this electron-bifurcating LCT to enable it to grow on lactate while producing acetate and butyrate.^18^ Considering the similarity in the bioenergetics of lactate and glycerol metabolism, a bifurcating enzyme analogous to LCT that couples glycerol and/or G3P oxidation with the oxidation of Fd_red_ and reduction of NAD^+^ would offer a solution to the energetics of glycerol metabolism. Herein we demonstrate that a bifurcating enzyme of this type is indeed found in a wide diversity of both bacteria and archaea.

Four families of electron bifurcating enzymes are currently known. All use Fd as an electron carrier and three of the four also use NAD­(H) and/or NAD­(P)H as the second and/or third redox carriers. Members of the electron transfer flavoprotein (EtfAB) family use NADH to reduce Fd by also coupling NADH oxidation to the reduction of a high potential acceptor, such as crotonyl-CoA,^1^ pyruvate^17^ or quinone.^19^ Two different families of enzyme couple the oxidation of both NADH and Fd to the reduction of NADP^+^ for biosynthesis and these are the NADH-dependent ferredoxin NADP transhydrogenase termed NfnSL, which has been characterized from *P. furiosus* ^20^ and NfnABC, which was purified from the related hyperthermophilic archaeon *Thermococcus sibiricus*.^21^ NfnSL is the only member of its family while NfnABC is part of a diverse family of bifurcating enzymes known as BfuABC, all of which utilize Fd and NAD­(P) but use a variety of compounds as the third redox carrier.^22^ The fourth bifurcating enzyme family known does not use NAD­(H) or NAD­(P)H and is represented by the methanogenic hydrogenase/heterodisulfide reductase complex MvhADG–HdrABC, in which the oxidation of hydrogen simultaneously reduces a high potential heterodisulfide and low potential Fd.^23^ Remarkably, although all four types of bifurcating enzyme use a flavin (FAD or FMN) to carry out the electron bifurcation, they are completely unrelated phylogenetically and differ in subunit composition and cofactor content.

Here we show that the hyperthermophilic archaeon, *Pyrococcus furiosus*, contains a member of a fifth electron bifurcating family that is involved in glycerol metabolism. Termed Ferredoxin-dependent NAD^+^ G3P dehydrogenase (FngABC), it is a homologue of the G3P dehydrogenase enzyme described in *T. maritima* and *A. woodii*. In FngABC, the energetically favorable oxidation of reduced Fd and reduction of NAD^+^ is coupled to the otherwise unfavorable oxidation of G3P coupled to NAD^+^ reduction. In essence, the exergonic oxidation of Fd drives the endergonic oxidation of G3P to DHAP with the concomitant reduction of NAD^+^. Electron bifurcation therefore provides a thermodynamic solution for anaerobic glycerol catabolism as the DHAP that is produced can directly enter the glycolytic pathway. Surprisingly, we show that FngABC and *E. coli* GlpABC are phylogenetically related and suggest that GlpABC may also be a bifurcating enzyme.

## Materials and Methods

### Purification of Recombinant *P. furiosus* FngABC

A linear PCR construct was designed to insert an FngABC overexpression cassette into the *P. furiosus* genome at the native *fngABC* gene location. The *fngABC* expression cassette, consisting of the 182 bp *slp* promoter,^24^ the 9× His-tag sequence (targeting insertion at the N-terminal of *FngA* gene PF2005), and the *pyrF* pop-out marker cassette containing 65 bp identical flanking regions for marker removal via homologous recombination,^24^ was added between ∼0.5 kb homologous flanking regions of the fngABC ATG start codon. The primers that were used are listed in Table S4. Each PCR product was obtained separately and the full linear ∼2.5 kb PCR construct was assembled via splicing by overlap extension and PCR.^25^ The FngABC PCR construct was transformed into *P. furiosus* COM1, as previously described.^26^ Genomic DNA of the transformants was isolated using the ZymoBead Genomic DNA Kit (Zymo Research) and isolates were screened by PCR, using primers that target outside of the homologous flanking regions. A *P. furiosus* strain containing the *fngABC* overexpression construct was sequence verified and designated as MW661.

Recombinant *P. furiosus* strains expressing tagged FngABC were grown on a 20 L scale as previously described.^27^ Cells were collected by centrifugation at 7,000 × *g* and stored at −20 °C until use. Cells (∼20 g) were lysed under strict anaerobic conditions by resuspension in 25 mM phosphate buffer, pH 7.5 in a Coy anaerobic chamber. Cell-free extracts were prepared by centrifuging at 100,000 × *g* for 1 h to remove particles and membrane fragments. The resulting supernatant was loaded onto a 5 mL HisTrap FF crude (Cytiva) and eluted with a gradient (0–0.5 M) of imidazole in 25 mM phosphate buffer, pH 7.5, containing 0.5 mM dithiothreitol according to manufacturer instructions. Fractions containing G3P to INT oxidation activity were pooled, 5-fold diluted with the phosphate buffer and loaded on 5 mL QHP HiTrap column (Cytiva). The enzyme was eluted with an increasing gradient (0–500 mM) of NaCl in 25 mM HEPES, pH 7.5, containing 0.5 mM cysteine. Active fractions judged pure by gel electrophoresis were pooled and stored at −80 °C until use. Anaerobic conditions were maintained throughout the purification procedures.

### Enzyme Assays

Dye-linked enzyme activities were measured anaerobically at 85 °C in a Cary 100 (Agilent) with 3-ml glass cuvettes sealed with rubber stoppers containing 50 mM HEPES, pH 7.5, 200 mM NaCl. G3P and NADH­(P)H oxidation were measured with iodonitrotetrazolium chloride (INT) at 500 nm (ε = 19.3 mM^–1^ cm^–1^) as the electron acceptor. Oxidation of G3P or reduction of DHAP was measured with NADH and NAD^+^ respectively at 340 nm (ε = 6.2 mM^–1^ cm^–1^). Specific activity (U mg^–1^) is defined as 1 μmol NAD­(P)­(H) or Glc-3P/DHAP reduced/oxidized per min/mg protein. Bifurcating assays were performed at 85 °C in a Cary 3500 (Agilent). For the bifurcating activity (eq 1), the reduction of Fd at 425 nm (ε = 13 mM^–1^ cm^–1^) was followed using 0.5 mM DHAP, 0.1 mM NADH and ∼35 μM Fd. The reverse (confurcating) reaction was measured by following the oxidation of Fd in which the Fd was first reduced with an equimolar amount of Ti-(III)-citrate before adding enzyme, 0.5 mM NAD^+^ and 0.5 mM G3P. Specific activity (U mg^–1^) is defined as 1 μmol Fd reduced/oxidized per min/mg protein.

### Structure Prediction

The structure of *P. furiosus* FngABC was predicted utilizing AlphaFold3.^28^ The [4Fe-4S] and [2Fe-2S] clusters were modeled into the FngC and FngA subunits of the apo AlphaFold 3 structure using a custom Python script. The script used the gemmi 0.7.5 and NumPy 2.4.2 packages. The donor clusters were extracted from PDB 5JFC (*P. furiosus* NfnSL). Each cluster was positioned by rigid-body Kabsch superposition onto the Cβ atoms of its coordinating cysteines (FngC Cys9/12/16/35; FngA Cys407/409/448/453). The Python script was coded with Anthropic’s Claude AI tools (Opus 4.6+; https://claude.ai/) and manually validated.

### Redox Titrations

Titrations were performed at 25 °C in a Cary 3500 spectrophotometer (Agilent) in 600 μL masked quartz cuvettes (Starna) anaerobically sealed with silicon stoppers. DHAP (Sigma), G3P (Sigma), NADH (GoldBio), NAD^+^ (Sigma), NADPH (Sigma), each at 5 mM final concentration, were anaerobically added from 5 mM stock solutions in small quantities with a 10 μL gastight syringe (Hamilton) to FngABC (∼5.5 μM in 400 μL) prepared in 50 mM HEPES, pH 7.5, with 200 mM NaCl. UV–visible spectra (200–800 nm) were recorded after each addition. All solutions were prepared under strict anaerobic conditions, and stock solutions of titanium citrate were prepared as described previously.^29^

### Phylogenetic Analysis

InterPro functional analysis was used provide targeted approach to finding the A subunit homologues. This was accomplished using the InterPro Domain Architectures web tool (IDA; https://www.ebi.ac.uk/interpro/search/ida/;^30^) in January 2026. After examining the InterPro signatures for *Pyrococcus* FngA and several characterized GlpA proteins, it was determined that a minimum of two InterPro domains were needed to find homologous proteins, approximately 500 amino acids in length. The first IPR007419, BFD-like [2Fe-2S]-binding domain, an uncharacterized domain often associated with small proteins that bind Fe. The second IPR006076, an FAD dependent oxidoreductase domain, is found in glycerol-3-phosphate dehydrogenase, sarcosine oxidase beta subunit, D-amino acid dehydrogenase, and other FAD dependent oxidoreductases. Using the IDA tool, nine domain architectures were extracted from InterPro, totaling more than 5,516 A subunit protein sequences. However, 95% (5,252) of the sequences were located in just one architecture and the representative amino acid length of 563 amino acids was the only one close to the target size. Thus, only these proteins were selected for further analyses.

The 5,252 A subunit sequences were aligned using Clustal Omega (version 1.2.4) with the default parameters.^31^ ClipKit was used with the “smart-gap” setting to remove insignificant gaps from the alignment,^32^ and the resulting alignment was used to construct a maximum likelihood phylogenetic tree using IQ-Tree (version 3.0.1;^33^ ). The IQ-Tree ModelFinder was used to automatically determine the best-fit model (i.e., Q.PFAM+I+R10). This model tree was tested using ultrafast bootstrap approximation (UFBoot) and Shimodaira-Hasegawa–like approximate likelihood ratio tests with 3,000 bootstrap replicates. The UFBoot trees were optimized by nearest neighbor interchange (NNI) to reduce overestimating branch supports and produce a consensus tree. iTOL (version 7.6;^34^) was used for display and analysis of the consensus phylogenetic tree without applying UFBoot cutoffs.

### Genome Context Analysis

To determine whether homologues of the *P. furiosus* FngA subunit where part of an FngABC enzyme, the 5,252 UniProt protein IDs were uploaded into the EFI-GNT (https://efi.igb.illinois.edu/efi-gnt/;^35,36^ ) genome neighborhood analysis tool. This tool removed about 979 sequences for various reasons, leaving 4,273 genome neighborhood diagrams with 10 proteins upstream and 10 downstream of the GlpA/FngA target protein. Each of the 21 genome neighborhood proteins have InterPro scan annotations, sequence length, nucleotide start/stop and strand direction. The EFI-GNT browser allowed us to visualize two distinct groups of organisms those with all three Fng subunits, FngABC, and those with all three Glp subunits, GlpABC.

This revealed that while FngB and GlpB show little sequence similarity, they do share IPR036188, a FAD/NAD­(P)-binding domain superfamily. Interestingly, they could also be distinguished from each other via using additional IPR numbers, IPR023753, a FAD/NAD­(P)-binding domain plus IPR051691, a Cyanogenesis, Opine Oxidation, G-3-P Dehydrogenation family for FngB and IPR009158, Glycerol-3-phosphate dehydrogenase, GlpB subunit family for GlpB. For FngC and GlpC there are no overlapping InterPro functions, making it them easy to distinguish from each other, FngC contains IPR036593, CPE0013-like superfamily (a domain of unknown function; DUF1667) and GlpC contains IPR017753, a glycerol-3-phosphate dehydrogenase or GlpC subunit family. In addition, a list of InterPro numbers were generated where the entry’s title contained use some form of the word glycerol (i.e., glycerol or glycero-). These InterPro numbers were collated into terms list from further analysis below (Table S5).

This data set was exported the ENF-GNT as SQLite database for quantitation. The database was loaded into the DB Browser for SQLite (https://github.com/sqlitebrowser/sqlitebrowser) application and a custom view of the data linking the neighbors and attributes tables together was created. This view was then exported from DB Browser to a CSV file. The CVS file was processed by a custom Python script (#1) into a transposed Microsoft Excel file for visual inspection. Transposed Excel file was analyzed by a custom Python script (#2) that searched for specific regular expression (regex) patterns of InterPro numbers (term list; Table S5) outputting metadata (e.g., accession numbers, sequence length, direction, match count, etc.) for each match (Table S6). Finally, another Python script (#3) analyzed the matches from script #2 to compile a list of probable complexes based on the protein matches intergenic distances (≤300 bps) and colocation to the A subunit (within 3 proteins up- or downstream). It also counted the number of proteins with regex matches of the associated terms based solely on colocation to the A subunit (within 10 genes up- or downstream). This final output file was combined with UniProt’s metadata to determine which A subunits were colocated in the same organism’s genome using the UniProt Proteome IDs.^37^ This combined file was reformatted for publication and used to annotate the phylogenetic tree above (Table S2). The Python scripts were coded or revised with Anthropic’s Claude AI tools (Opus 4.6+; https://claude.ai/) and manually validated.

## Results and Discussion

The hyperthermophilic archaeon *P. furiosus* grows well on a variety of polysaccharides, such as starch and lamarin, and disaccharides, such as maltose and cellobiose. Although the organism is not able to utilize monosaccharides, such as glucose,^38^ its genome contains a gene cluster that suggests it is able to metabolize glycerol. Specifically, the cluster contains five genes within an operon structure that are annotated as encoding glycerophosphodiesterase (PF2003), glycerol kinase (PF2004), FAD-containing glycerol-3-phosphate (G3P) dehydrogenase (PF2005), an FAD-dependent oxidoreductase (PF2006) and a DUF1667 domain-containing protein (PF2007) (Figure S1). Although transcriptomic analyses have shown that all five genes are coexpressed during growth on disaccharides and peptides, both with and without elemental sulfur,^39,40^ our attempts to grow *P. furiosus* on glycerol as the primary carbon source, with and without elemental sulfur, were unsuccessful. In a previous report, homologues of the genes PF2005-PF2007 from the related hyperthermophilic archaeon *Thermococcus kodakarensis* (TK1396-TK1398), were heterologously expressed together and individually in *E. coli*. The resulting heterotrimeric complex was shown to oxidize G3P using an artificial tetrazolium dye but not NAD^+^ as the electron acceptor. It was referred to as anaerobic G3P dehydrogenase (G3PDH^41^). However, it was also shown that *T. kodakarensis* does not grow on glycerol and the function of its G3PDH was therefore unknown.

Based on these prior results, the initial goal of the present work was to characterize the putative G3PDH from *P. furiosus* and to determine its physiological electron carrier and metabolic function. A recombinant *P. furiosus* strain was constructed wherein the native gene cluster PF2005-PF2007 was modified to use the strong promoter that drives the expression of the gene encoding the S-layer protein (PF1399) with a His_9_-tag encoded at the N-terminus of the protein encoded by PF2005 (Figure S1). The recombinant enzyme that was produced was purified from a cytoplasmic extract by affinity and anion exchange chromatography yielding a heterotrimer by SDS-gel electrophoresis corresponding to the predicted masses of 54.8, 45.4, and 12.7 kDa for PF2005, PF2006 and PF2007, respectively, with a molecular weight for the heterotrimer of 112.9 kDa. The yield of the purified enzyme was ∼1 mg/g wet cell paste. As was found for the *T. kodakarensis* enzyme, the enzyme from *P. furiosus* oxidized G3P and reduced the artificial dye iodonitrotetrazolium chloride (INT) but it did not reduce NAD^+^ or NADP^+^ (Table 1). However, the enzyme did oxidize NADH but not NADPH and reduce INT (Table 1) showing that it was able to use NAD­(H) as an electron carrier.

**Table 1:** Reactions Catalyzed by *P. Furiosus* FngABC

Reaction	Electron carrier measured	Specific Activity (U/mg)
DHAP + Fd_ox_ + 2NADH	Fd	5.0 ± 0.7
Fd_red_ + NAD^+^ + G3P	Fd	2.5 ± 0.7
DHAP + NADH	NADH	<0.01
G3P + NAD^+^	NADH	<0.01
G3P + Fd_ox_	Fd	<0.01
NADH + Fd_ox_	Fd	<0.01
DHAP + Fd_red_	Fd	<0.01
NADH + O_2_	NADH	19.2 ± 4.4
G3P + INT	INT	15.5 ± 1.5
NADH + INT	INT	75.3 ± 7.2
NADPH + INT	INT	0.19 ± 0.06

Analysis of their amino acid sequences indicated that PF2005 contains a flavin and a [2Fe-2S] cluster, PF2006 contains a flavin and PF2007 contains a [4Fe-4S] cluster (Figure 1). Such an arrangement of redox cofactors is reminiscent of those found in electron bifurcating enzymes such as the NADH-dependent ferredoxin NADP transhydrogenase (NfnSL) of *P. furiosus* (Figure 1). In NfnSL, the thermodynamically unfavorable reduction of NADP^+^ (E′ −380 mV under physiological conditions) by NADH (E′ −280 mV) is driven by coupling it to the oxidation of reduced Fd (E′ −500 mV).^20,42^ Consequently, we showed that this recombinant heterotrimer (PF2005–2007) of *P. furiosu* *s* is, indeed, an electron bifurcating enzyme and its activity is dependent on the presence of all three substrates, Fd, G3P (DHAP) and NAD­(H) (Figure S2). Specifically, it catalyzed the DHAP and NADH-dependent reduction of oxidized *P. furiosus* Fd and the G3P and NAD^+^-dependent oxidation of reduced Fd (Table 1 and Figure S2), according to eq 1 (where Fd is a one electron carrier).

1 $${\mathrm{G3P}\;+\;\mathrm{2 NAD}}^{+}{\;+\;\mathrm{2 Fd}}_{\mathrm{red}}\;↔{\;\mathrm{DHAP}\;+\;\mathrm{2 NADH}\;+\;\mathrm{2 Fd}}_{\mathrm{ox}}$$

**Figure 1:**
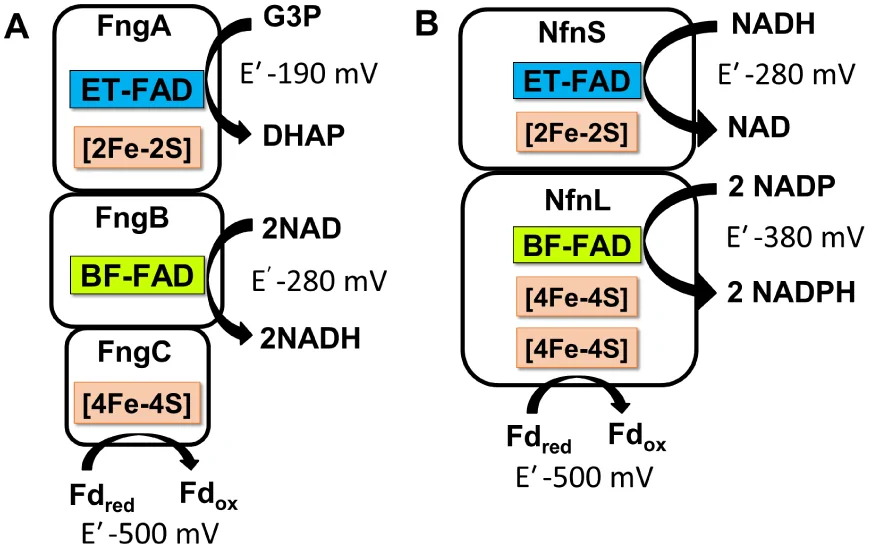
Comparison of FngABC and NfnSL. Cofactor contents and proposed electron bifurcation reactions are shown for A) *P. furiosus* FngABC, B) *P. furiosus* NfnSL.^20^ The effective potentials (E′) for each half reaction under physiological conditions are indicated.

The bifurcating and confurcating activities of the recombinant *P. furiosus* enzyme were comparable showing that the bifurcation reaction (eq 1) is readily reversible (Table 1). We therefore propose to name this enzyme Ferredoxin-dependent NAD^+^: Glycerol-3-phosphate oxidoreductase (FngABC). The bifurcating activities of FngABC were similar to the nonbifurcating NADH dye-dependent activity (Table 1). The enzyme also oxidized NADH and reduced O_2_ in a nonbifurcating reaction, but since *P. furiosus* is an obligate anaerobe, this is a nonphysiological reaction. Notably, FngABC did not reduce Fd or NAD^+^ using G3P as the electron donor, nor did it oxidize reduced Fd or NADH with DHAP as the electron acceptor showing that the bifurcation reactions are tightly coupled. FngABC is also specific for NAD­(H) as it did not show significant activity with NADP­(H) in place of NAD­(H) (Table 1).

Since *P. furiosus* and *T. kodakarensis* ^41^ do not utilize glycerol as a carbon source, we speculate that the glycerol that serves as the substrate for FngABC is derived from membrane lipids during turnover as part of normal metabolism. This is supported by the presence of a glycerophosphodiester phosphodiesterase homologue in the FngABC gene cluster (Figure S1), and is consistent with expression of the enzyme under all growth conditions.^39,40^ However, little is known about the turnover of membrane lipids in Archaea.^43,44^ Furthermore, *P. furiosus* does not appear to encode a glycerol facilitator (GlpF-like) as the case with *T. maritima* and *A. woodii*, anaerobic bacteria that do grow with glycerol as the sole carbon source.^15,16^

The predicted structure of FngABC lacking iron sulfur clusters was determined by AlphaFold3 ^28^ and a custom Python script was used to insert the clusters (Figure 2). A prior study of the homologous enzyme from *T. kodakarensis* ^41^ showed that the equivalent of the FngA subunit in isolation oxidized G3P and reduced the INT dye and so *P. furiosus* FngA is assumed to bind G3P/DHAP and this is shown in the structural model (Figure 1). As shown in Figure 1, the predicted cofactor content of FngABC from amino acid sequence analyses is analogous to that of the well-characterized bifurcating enzyme *P. furiosus* NfnSL, in which a central FAD binds the midpotential acceptor (NADP^+^).^20^ In NfnSL this so-called bifurcating FAD (BF-FAD) accepts electrons both from the high potential donor (NADH), which is oxidized by a second electron-transferring FAD (ET-FAD) via a [2Fe-2S] cluster, forming the high potential electron pathway, and from the low potential donor (Fd), which is oxidized via two [4Fe-4S] clusters, forming the low potential pathway (Figure 1). We therefore propose that FngB contains the BF-FAD that receives electrons from the high potential donor (G3P) that is oxidized by the ET-FAD in FngA which, with a [2Fe-2S] cluster, forms the high potential electron pathway and from the low potential donor (Fd) that is oxidized by the [4Fe-4S] cluster in FngC that forms the low potential pathway (Figure 1).

**Figure 2:**
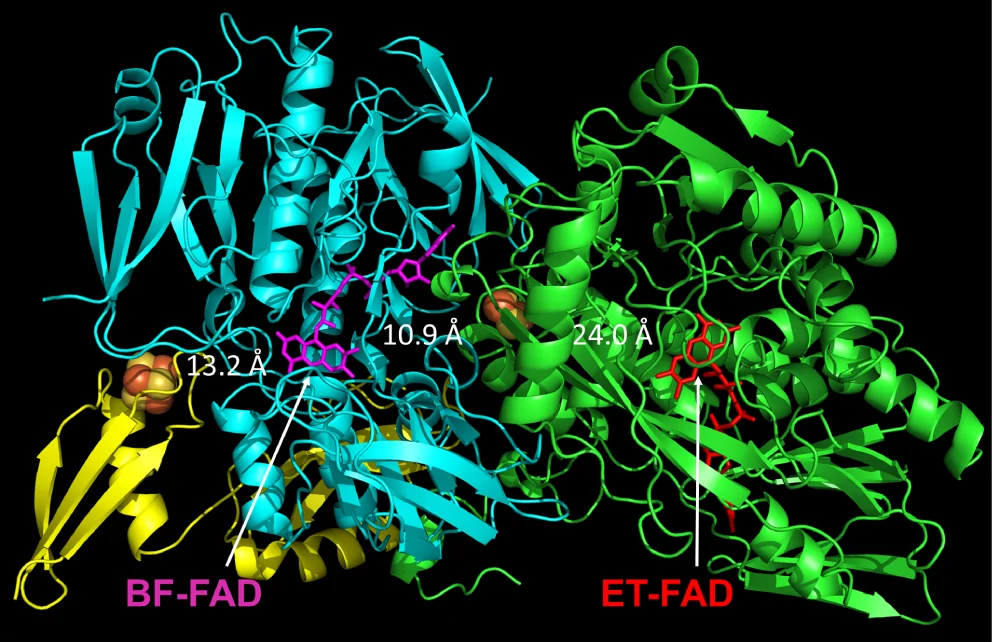
Predicted structure of *P. furiosus* FngABC. The sequences of *P. furiosus*FngABC (PF2005 - PF2007) were subjected to structural prediction using the AlphaFold3 platform^28^ and a custom script coded by Antropic’s Claude AI tools (Opus 4.6+; https://claude.ai/). FngA (PF2005) is depicted in green, FngB (PF2006) is depicted in cyan and FngC (PF2007) is depicted in yellow. The iron and sulfur atoms of the clusters are indicated as orange and yellow spacefill, respectively, and the bifurcating (BF-FAD, red) and electron transfer (ET-FAD, magenta) flavins are indicated. The distances between the cofactors are indicated in white text.

The predicted structure of FngABC (Figure 2) shows that the [4Fe-4S] and [2Fe-2S] clusters are within electron transfer distance of the BF-FAD (≤14 Å) but the [2Fe-2S] and the ET-FAD are separated by ∼24 Å in the FngA subunit. This suggests that a significant confirmational change must occur to bring these two cofactors within electron transfer distance, or it may reflect the inability of AlphaFold to accurately model an enzyme containing redox cofactors. In any event, under physiological conditions, it is proposed that FngABC converts G3P to DHAP and reduces NAD^+^, a reaction driven by the simultaneous electron transfer from Fd_red_ to NAD^+^, based on the reaction mechanism established by structural and spectroscopic analyses of NfnSL (Figure 1).^20^ Remarkably, however, there is no sequence homology between the subunits of FngABC and those of NfnSL indicating that these enzymes evolved independently, nor is there any sequence similarity of FngABC with any of the subunits of any member of the other three families of bifurcating enzyme (BfuABC, EtfAB and HdrABC). Hence, *P. furiosus* FngABC represents the prototypical member of a new fifth class of bifurcating enzyme.

### Spectroscopic Properties of FngABC

FngABC as purified exhibited a UV–visible spectrum (Figure 3) with characteristic absorption at 450 and 370 nm for flavin with a broad contribution in the 300 to 500 nm region for the iron sulfur clusters showing that the enzyme is in the oxidized state. Stepwise reduction with the artificial reductant Ti­(III)-citrate revealed that both the flavins and the clusters are reduced but, unlike the situation with *P. furiosus* NfnSL, no intermediate flavin semiquinone (ASQ or NSQ) was observed during the titration.^20^ Titration of FngABC with the high-potential substrate G3P led to reduction of ∼50% of the total flavin, consistent with the reduction of the ET-FAD in FngA subunit, but without the formation of an observable flavin semiquinone (Figure 3). This is once more in contrast to NfnSL where upon titration with its high-potential substrate NADH, a clear NSQ optical signature around 620 nm is observed.^20^ The titration of FngABC with its midpotential carrier NADH shows the reduction of most of the flavin indicating that electrons from NADH can reduce both the BF-FAD in the B-subunit and ET-FAD in the A-subunit but again, without an observable flavin semiquinone, in contrast to the situation with NfnSL when titrated with its midpotential donor NADPH (Figure 3).^20^

**Figure 3:**
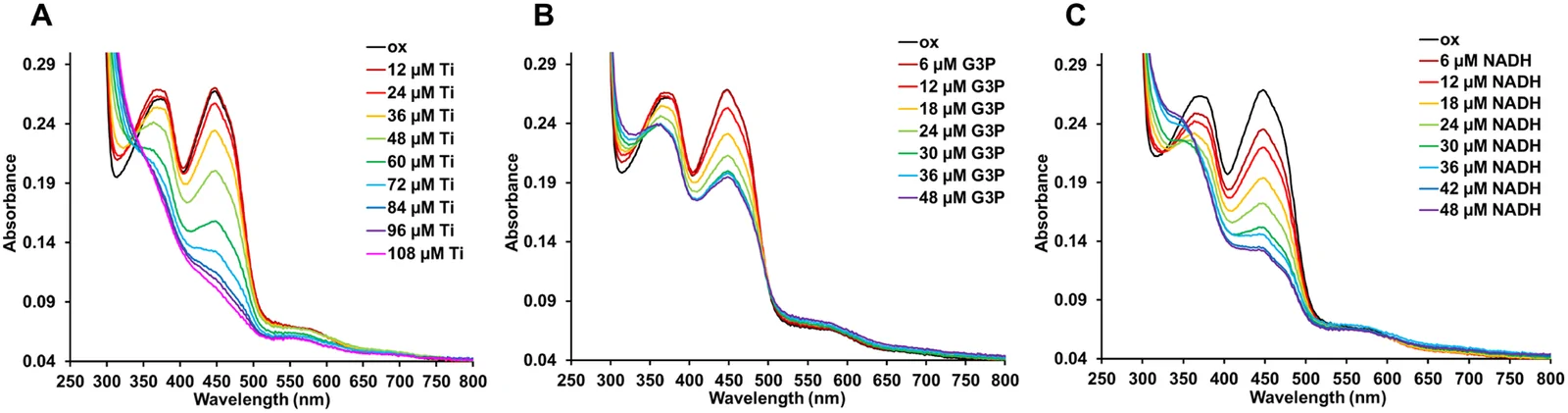
Redox titrations of *P. furiosus* FngABC. A. Titration with the low potential chemical reductant Ti­(III) citrate B. Titration with the high potential substrate G3P. C. Titration with the mid potential substrate NADH. See text for details.

Both FAD moieties in NfnSL give rise to semiquinones during catalysis, however, only the ET-FAD displays a stable NSQ signal while the BF-FAD OX/SQ/HQ potentials are highly crossed and only a very short-lived ASQ signal is generated.^45,46^ In FngABC, when the ET-FAD in FngA is reduced with G3P, the flavin should reduce the adjacent [2Fe-2S] cluster and form a stable semiquinone analogous to NfnSL but G3P is unlikely to reduce BF-FAD via the [2Fe-2S] cluster considering the thermodynamic barrier imposed by a highly crossed BF-FAD.^45^ It is therefore not clear how FngABC bifurcates electrons with the lack of observable flavin semiquinones in our titrations and the bifurcating mechanism and control of electron flow likely differs from that established in NfnLS (Figure 1).^20^ Clearly, further analyses using other spectroscopic approaches such as direct electrochemistry and EPR and transient absorption spectroscopy^20^ are needed to resolve this issue.

Previous studies have established that the BF-FAD of NfnSL has highly crossed potentials and that this is in part determined by a highly conserved Arg residue interacting with N5 site of the BF-FAD.^20,47^ A similar situation exists with the BF-FAD in the unrelated bifurcating enzyme Fix/EtfABCX and mutation of that Arg abolished bifurcation activity and associated electron transfer.^48^ However, inspection of the BF-FAD in FngB did not reveal a corresponding Arg residue in the vicinity of the BF-FAD (Figure S3). This may be related to the absence of an observable stable SQ species during the titrations of FngABC described above. Moreover, in all of the 1,485 homologues of *P. furiosus*FngABC described below, the equivalent of the conserved Arg in the other enzymes is replaced by a conserved Gln-Cys-Ile-His motif (Figure S3). This supports the notion that the mechanism of this fifth class of electron bifurcating enzyme represented by FngABC is, indeed, different from the well-established mechanisms of both NfnSL^20^ and Fix/EtfABCX.^48^

### Distribution and Phylogeny of FngABC Homologues

Anaerobic bacteria previously studied that utilize glycerol as a carbon source, including *T. maritima* and *A. woodii*, as well as *Thermoanerobacter wiegelii* and *Thermoanerobacter brockii*, all contain close homologues of *P. furiosus* FngABC.^15,16,49^ The FngABC operon in all of these organisms also includes a gene encoding glycerol kinase, like that of *P. furiosus* (Figure S1). We therefore predict that these FngABC enzymes also carry out the same electron bifurcation reaction. For example, this would explain the very low activity (32 mU/mg) of the nonbifurcating G3P oxidation reaction with NAD^+^ as the electron acceptor measured in cell extracts of the acetogen *A. woodii*.^15^

The A subunit of *P. furiosus* FngABC gene is also highly homologous to the A-subunit of the previously characterized “anaerobic” G3P dehydrogenase termed GlpABC from *E. coli*.^50–52^ This enzyme enables glycerol utilization under anaerobic conditions with fumarate as the electron acceptor in which electrons from G3P oxidation enter the quinone pool. However, the *E. coli* GlpBC subunits show no relationship to the FngBC subunits and thus the quinone-dependent GlpABC complex is clearly distinct from the ferredoxin-dependent FngABC. This is expected since *P. furiosus* lacks a quinone-based respiratory system and *E. coli* lacks the conventional Fd found in fermentative anaerobes. Nevertheless, we hypothesize that *E. coli* GlpABC is, indeed, a bifurcating enzyme. Hence, by homology with FngA, GlpA contains a 2Fe-cluster and FAD and oxidizes G3P to DHAP. From sequence analysis we predict that GlpB contains an NAD^+^-reducing ET-FAD while GlpC has two [4Fe-4S] clusters and two noncubane iron–sulfur clusters with the 2xCCG signature (IPR004017) that could potentially reduce quinone. The 2xCCG noncubane cluster was first identified in the electron bifurcating HdrABC-MvhAGD complex in methanogens^53^ (Figure S4). Hence, by analogy with FngABC and NfnSL, we propose *E. coli* GlpABC couples G3P oxidation to DHAP (E_o_′ −190 mV) to the exergonic reduction of quinone (E_o_′ ∼ −80 mV) and the endergonic reduction of NAD^+^ (E′ −280 mV) (see Figure S4).

*E. coli* GlpABC was first characterized over 40 years ago although only the GlpAB subcomplex was purified and GlpC was not observed.^51^ It was subsequently shown that the enzyme consists of three subunits GlpABC where GlpC is membrane associated and during purification of GlpAB the GlpC subunit was identified in the detergent insoluble fraction.^50,52^ Expression of all three subunits of GlpABC are required for anaerobic growth on glycerol with fumarate as the electron acceptor.^50^ Even though there is no evidence from its sequence that GlpC is membrane associated, such a location would provide a link to quinone reduction and anaerobic respiration. Hence, we propose that *E. coli* GlpABC is a bifurcating enzyme that reduces oxidizes G3P and simultaneously reduces NAD^+^ and quinone (Figure S4). However, demonstrating that experimentally will be very challenging due to the membrane association of the enzyme and that is beyond the scope of this study. In fact, quinone-linked bifurcation could not be demonstrated with the membrane-associated bifurcating enzyme Fix/EtfABCX.^54^ Instead, substrate-dependent reduction of iron–sulfur clusters within the purified ABCX holoenzyme monitored by EPR spectroscopy was used as a proxy for bifurcation.^48^ Such an approach may be useful with *E. coli*, assuming the GlpABC holoenzyme can be obtained.

Many halophilic Archaea also contain a homologue of *E. coli* GlpABC and that is essential for glycerol utilization as shown for the model haloarchaeon *Haloferax volcanii* (*Hvo*).^13^ We propose that this enzyme also carries out an electron bifurcation reaction (Figure S4) coupling G3P oxidation to respiration via quinone reduction. On the other hand, an “aerobic” quinone-reducing heterodimeric G3P dehydrogenase was also recently characterized from the thermoacidophilic archaeon *Sulfolobus acidocaldarius*.^3^ Neither of its two subunits contained iron sulfur clusters nor were they homologous to any of the *E. coli* GlpABC subunits. Instead, the enzyme was a homologue of the nonbifurcating G3P dehydrogenase of *E. coli* termed GlpD. The membrane associated *S. acidocaldarius* enzyme therefore does not catalyze an electron bifurcation reaction and is not part of the FngA/GlpA family represented by the *P. furiosus* and *E. coli* enzymes, as discussed below.

A phylogenetic analysis of the A-subunits in Glp-like G3PDHs was recently carried out^3^ but at the time it was not evident that some of these enzymes might carry out electron bifurcation reactions. We therefore carried out a similar analysis using 5,252 homologues of the *P. furiosus* FngA subunit extracted from the UniProt database^55^ along with genome context analysis of 20 surrounding proteins from 4,273 of the 5,252 FngA homologues. This revealed that 35% (1,485) of the FngA homologues are also associated with the homologues of *P. furiosus* FngBC and therefore all of these heterotrimeric enzymes are proposed to be bifurcating (Figure 4). Most are found in bacteria and mainly within the Clostridia (Table S1). Some species contain multiple FngABC homologues, including lactate-utilizing *A. woodii* ^15^ and the gut microbe *Eubacterium limosum*,^18^ which contain four and eight homologues, respectively (designated *Aw*1–4 and *El*1–8 in Figure 4). Indeed, many FngABC enzymes appear to utilize polyols other than glycerol because none of the 20 genes surrounding their FngA had InterPro entries that were related to glycerol and many contained genes encoding sugar kinases and other polyol-metabolizing enzymes (Table S2).

**Figure 4:**
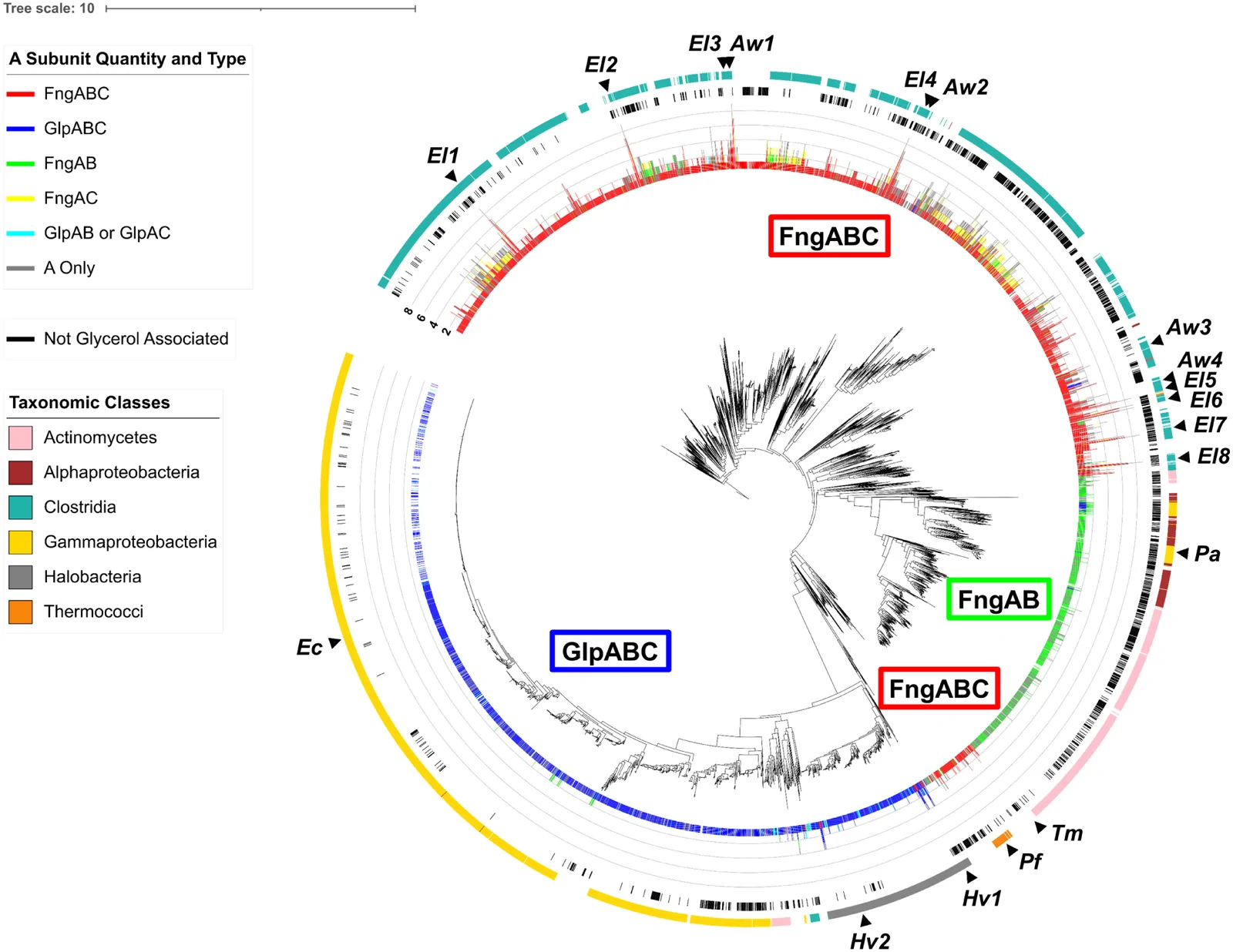
The phylogenetic distribution of *P. furiosus*FngA homologues and their genome neighborhood associations. The central tree shows the phylogenetic relationship of 5,252 FngA homologues based on two InterPro domains (IPR006076 and IPR007419). The surrounding rings provide details on the FngA homologues (4,273 of the 5,252) and their genome context associations. Ring 1 (innermost): a stacked bar chart with four gray grid lines, labeled 2, 4, 6, and 8, from inner to outer indicating the number of FngA homologues within the same organism (using UniProt Proteome ID). The bar’s color shows the predicted complex (ABC, AB, AC or A only) in that proteome. Proteomes with multiple homologues have identical bar signatures because they are repeated for each homologue in the ring (e.g., El1–8). Ring 2: A single ring of black bars shows that none of the 20 genes surrounding FngA (minus the complex subunits, if present) in the genome have InterPro entries that are related to glycerol. Ring 3 (outermost): A single ring of multicolored bars that show the most abundant (>3%, plus Pfu) taxonomic classes where FngABC homologues are found. Black triangles show key organisms with FngA homologues abbreviated as *Aw,* *Acetobacterium woodii* *; Ec*, *Escherichia coli* *K12; El,* *Eubacterium limosum* *; Hv,* *Haloferax volcanii* *; Pa,* *Pseudomonas aeruginosa* *; Pf,* *Pyrococcus furiosus*, and *Tm,* *Thermotoga maritima*. For *El* and *Aw,* the number indicates how many FngA homologues they contain and these are numbered clockwise around the tree.

Closely related to these bacterial FngABCs are FngA homologues that are associated with FngB but not with FngC (540 or 13% of the total, Table S1), and these are designated FngAB in Figure 4. In addition, 4% (175) of the FngAs have an FngC without an obvious FngB nearby. In the absence of FngC it seems unlikely that the FngAB enzymes are able to bifurcate and potentially they catalyze the energetically favorable reverse reaction, oxidation of NADH to reduce the keto sugar phosphate to the hydroxy form. These nonbifurcating FngAB homologues are found predominantly in Actinomyces and also in some Alpha- and Gammaproteobacteria (Figure 4). Phylogenetically separated from the bacterial FngABCs is a small group that includes *P. furiosus* FngABC and other members of the hyperthermophilic archaea (51 in total, Table S1) such as *T. kodakarensis*. These are designated as the second FngABC group in Figure 4. Also included in this second group of FngABCs are enzymes from the thermophilic bacterial class Thermotogae (46 in total). The hyperthermophile *T. maritima* is the prototypical member and is known to contain a high proportion of archaeal-related genes.^56^ FngABC can now be included among them given its phylogenetic position (Figure 4).

As shown in Figure 4, other archaea homologues of *P. furiosus* FngA are those in the halophilic archaea (261 or 5% of the total; Table S1). Most of them contain a single homologue with the well-studied *H. volcanii* being a notable exception as it has two (designated *Hv*1 and *Hv*2 in Figure 4). However, these halophiles do not contain homologues of *P. furiosus* FngBC, rather, they are associated with homologues of *E. coli* GlpBC. These are part of the 1,628 FngA homologues (38% of the total; Table S1) that are true homologues of *E. coli* GlpABC and designated as such in Figure 4. Only a few of these GlpA-like homologues lack either GlpB or GlpC (118, or 3% of the total; Table S1). We propose that all of these trimeric enzymes are also bifurcating, membrane-associated and quinone-reducing and linked to respiratory processes. Besides the halophilic archaea, these GlpABC enzymes are found almost exclusively in the Gammaproteobacteria with that of *E. coli* being the prototypical member (Figure 4).

FngABC (1,485) and GlpABC (1,628) enzymes together contain 73% of the 4,273 *P. furiosus* FngA homologues where genome context is available and all are proposed to carry out electron bifurcation (Table S1). Two features distinguish them from each other. First, a significant fraction of the FngABC enzymes (32%) are not associated with genes related to glycerol metabolilsm and they are assumed to oxidize other polyol derivatives. Far fewer of the GlpABC enzymes (13%) were not associated with glycerol, indicating that vast majority (87%) do indeed utilize only G3P. Accordingly, 14% of the microbes that contain FngABC enzymes have one or more additional FngABC homologue, while only 0.8% of the GlpABC-containing organisms have more than one GlpABC homologue. The phylogenetic and the genome context analyses combined also show that the FngA and GlpA related complexes diverged into various clades very differently (Figure 4). FngA related clades have long branch lengths indicating more diverse A subunits, while the GlpA groups have much shorter branch lengths suggesting a more conserved grouping and possibly a single function. In any event, we conclude that of those organisms that contain a homologue of the *P. furiosus* FngA subunit, 73% of them are likely electron bifurcating enzymes, with 35% homologous to the *P. furiosus* holoenzyme FngABC and 38% homologous to the *E. coli* GlpABC holoenzyme.

Our discovery of a bifurcating FngABC addresses the long-standing inconsistency in anaerobic glycerol metabolism in which the oxidation of G3P coupled to the reduction NAD^+^ is thermodynamically unfavorable. In FngABC this reaction is simultaneously coupled to the exergonic oxidation of reduced Fd. The DHAP that is produced then enters the glycolytic pathway. As shown in Figure 5, reduced Fd for FngABC is produced by the oxidation of pyruvate catalyzed by pyruvate ferredoxin oxidoreductase (POR). With the FngABC system the oxidation of glycerol to acetate and CO_2_ yields 3 equiv of NADH and 2 ATP (eq 2). NADH can be used for biosynthesis or oxidized by pathways such as acetogenesis or reverse electron transport to balance the energetics of anaerobic metabolism.^15,16^

2 $$\mathrm{Glycerol}\;+\;\mathrm{3 NAD}\;+\;\mathrm{2 ADP}\;+\;\mathrm{2 Pi}\;\to {\;\mathrm{Acetate}\;+\;\mathrm{CO}}_{2}\;+\;\mathrm{3 NADH}\;+\;\mathrm{2 ATP}$$

**Figure 5:**
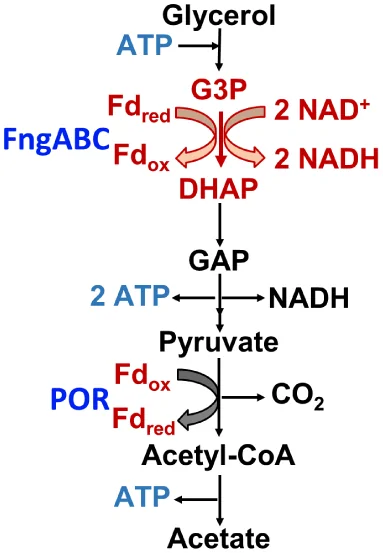
Schematic representation of glycerol metabolism in anaerobes utilizing FngABC. Glycerol is phosphorylated by glycerol kinase and G3P is oxidized by FngABC yielding DHAP, which further metabolized in the glycolytic pathway. The reduced Fd used to drive the FngABC reaction is produced through the oxidation of pyruvate by pyruvate Fd oxidoreductase (POR).

## Conclusions

We have shown that FngABC from *P. furiosus* is part of a new fifth family of electron bifurcating enzyme that is evolutionary distinct from the other four previously characterized families. The enzyme enables nonrespiratory anaerobes to efficiently oxidize glycerol to acetate and generate both NADH and ATP for biosynthesis. Interestingly, the FngABC family members contain a minimalist cofactor content of a [2Fe-2S] and a [4Fe-4S] cluster with two FADs. The centrally located NAD^+^-reducing BF-FAD receives high potential electrons through a [2Fe-2S] cluster next the G3P-oxidizing ET-FAD and low potential electrons from reduced Fd via a [4Fe-4S] cluster (Figure 1). This is simplest arrangement of cofactors known for any bifurcating enzyme and therefore will provide an advantage in interpreting the results of the spectroscopic analyses, such as from using direct electrochemistry and EPR and transient absorption spectroscopy, needed for what appears to be a mechanism of electron bifurcation that differs from that in the other four familes of bifurcating enzyme.

## Supplementary Material













## Notes

Figures S1–S4 and Table S4 (PDF)
Tables S1 (XLSX)
Tables S2 (XLSX)
Tables S3 (XLSX)
Tables S5 (XLSX)
Tables S6 (XLSX)

## Abbreviations

FngABC: Ferredoxin-dependent NAD^+^: glycerol-3-phosphate oxidoreductase
FnpABC: Fd-dependent NAD^+^: polyol oxidoreductase
G3P: glycerol-3-phosphate
DHAP: dihydroxyacetone phosphate
DHA: dihydroxyacetone
INT: iodonitrotetrazolium chloride
Fd: ferredoxin
BF-FAD: bifurcating flavin
ET-FAD: electron transfer flavin
ASQ: anionic semiquinone
NSQ: neutral semiquinone
GlpABC: anaerobic glycerol-3-phosphate dehydrogenase.
