# Supplementary material for: A New Family of Electron-Bifurcating Enzymes Represented by Ferredoxin- and NAD-Dependent Glycerol-3-Phosphate Oxidoreductase
Source: Biochemistry. 2026 Oct 7;65(20):3247–57. doi: 10.1021/acs.biochem.6c00512 (PMC13689815; doi:10.1021/acs.biochem.6c00512)
Supplement: Supplementary file 1 [file bi6c00512_si_001.pdf]

A New Family of Electron Bifurcating Enzyme Represented by Ferredoxin- and NAD-Dependent Glycerol-3-Phosphate Oxidoreductase

Gerrit J. Schut, Farris L. Poole and Michael W. W. Adams*

Department of Biochemistry & Molecular Biology, University of Georgia, Athens, GA 30602, USA

***Contact Information**

Michael W. W. Adams, Corresponding Author (adamsm@uga.edu)

Gerrit J. Schut (gerti@uga.edu)

Farris L. Poole (fpoole@uga.edu

Supplementary Information

Supplementary Figures S1-S4

Supplementary Tables S1-S6

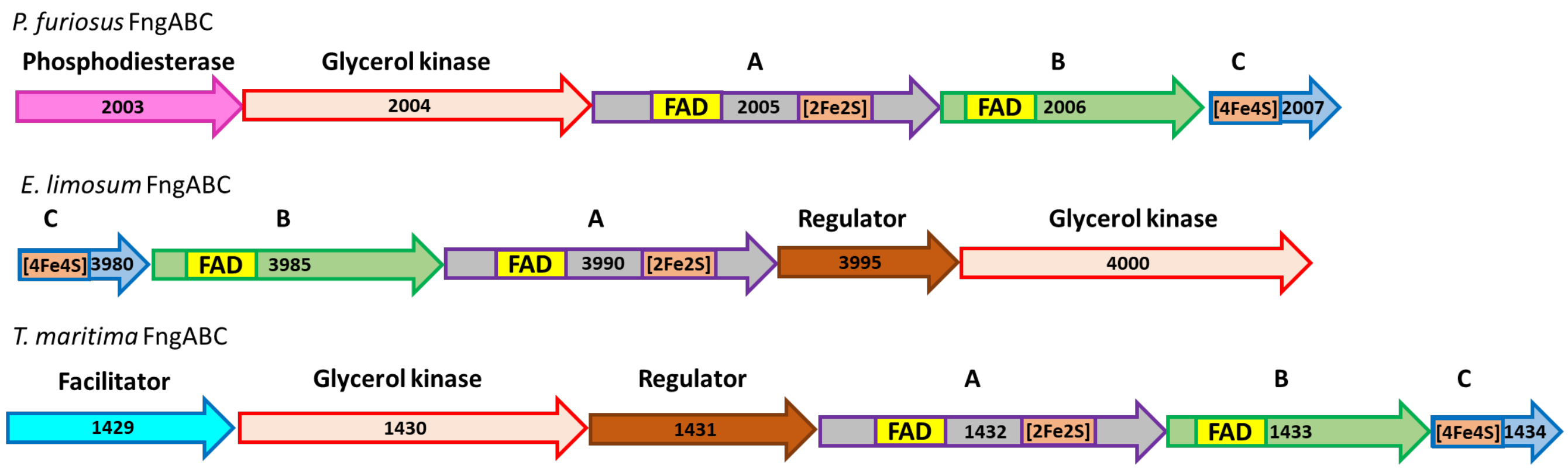

Figure S1. FngABC genome organization in *P. furiosus*, *T. maritima* and *A. woodii*. Gene clusters are not drawn to a precise scale and cofactor content is indicated with [2Fe2S], [4Fe4S] and FAD. ORF numbers are indicated inside the gene marker; *P. furiosus*, PF2003-2007; *E. limosum*, B2M23_13980-14000; *T. maritima*, TM1429-1434.

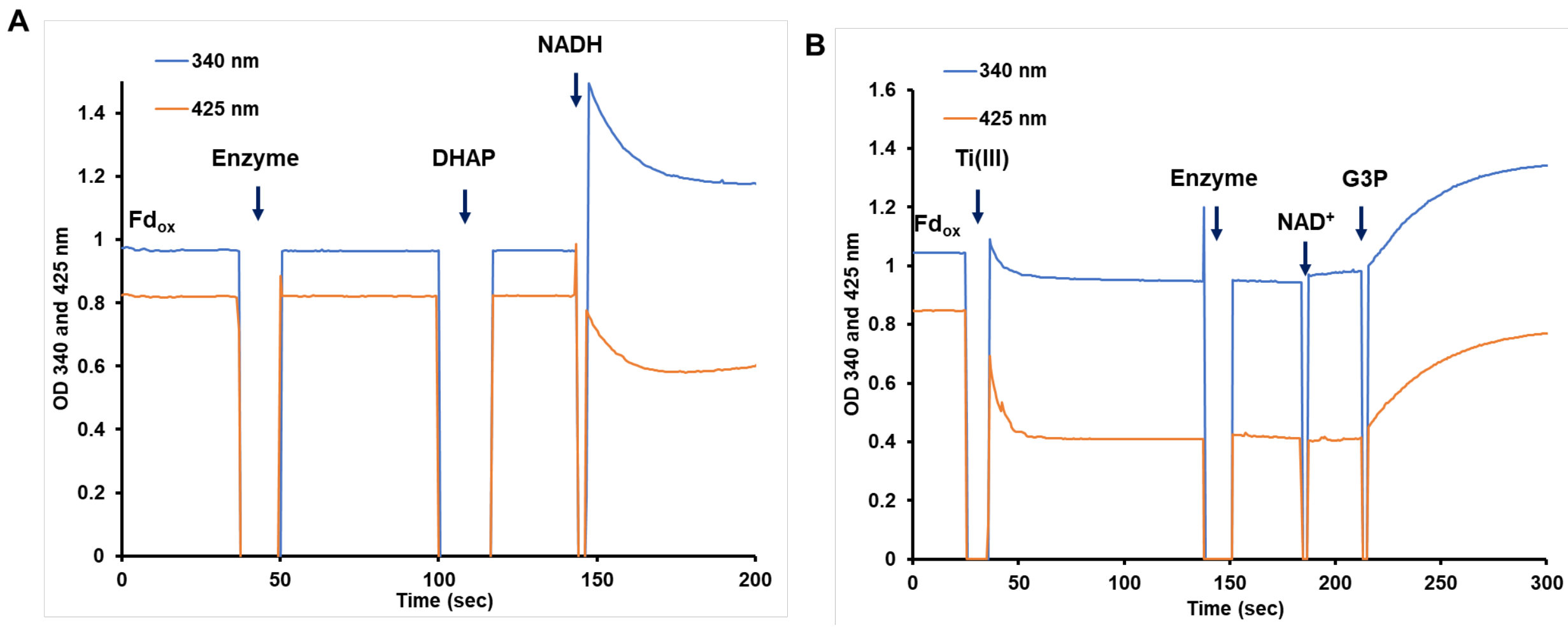

Figure S2. **Fd- and NAD/H-dependent FngABC reactions.** Reactions with purified *P. furiosus* FngABC were carried out in 400 µl anaerobic cuvettes by following the reduction or oxidation of Fd at 425 nm (orange line) and oxidation or reduction of NAD/H at 340 nm (blue line). The presence of Fd is responsible for the absorbance at the beginning of the reaction and oxidation or reduction of Fd also contributes to absorbance at 340 nm. Addition of each reaction component is indicated by arrows hence the transient decrease in the absorbance when the cuvette is removed from spectrophotometer. **A.** DHAP- and NADH- dependent reduction of Fd_{ox} **B.** G3P- and NAD^+-dependent oxidation of Fd_{red} (Fd_{ox} is first reduced with an equivalent of Ti(III).

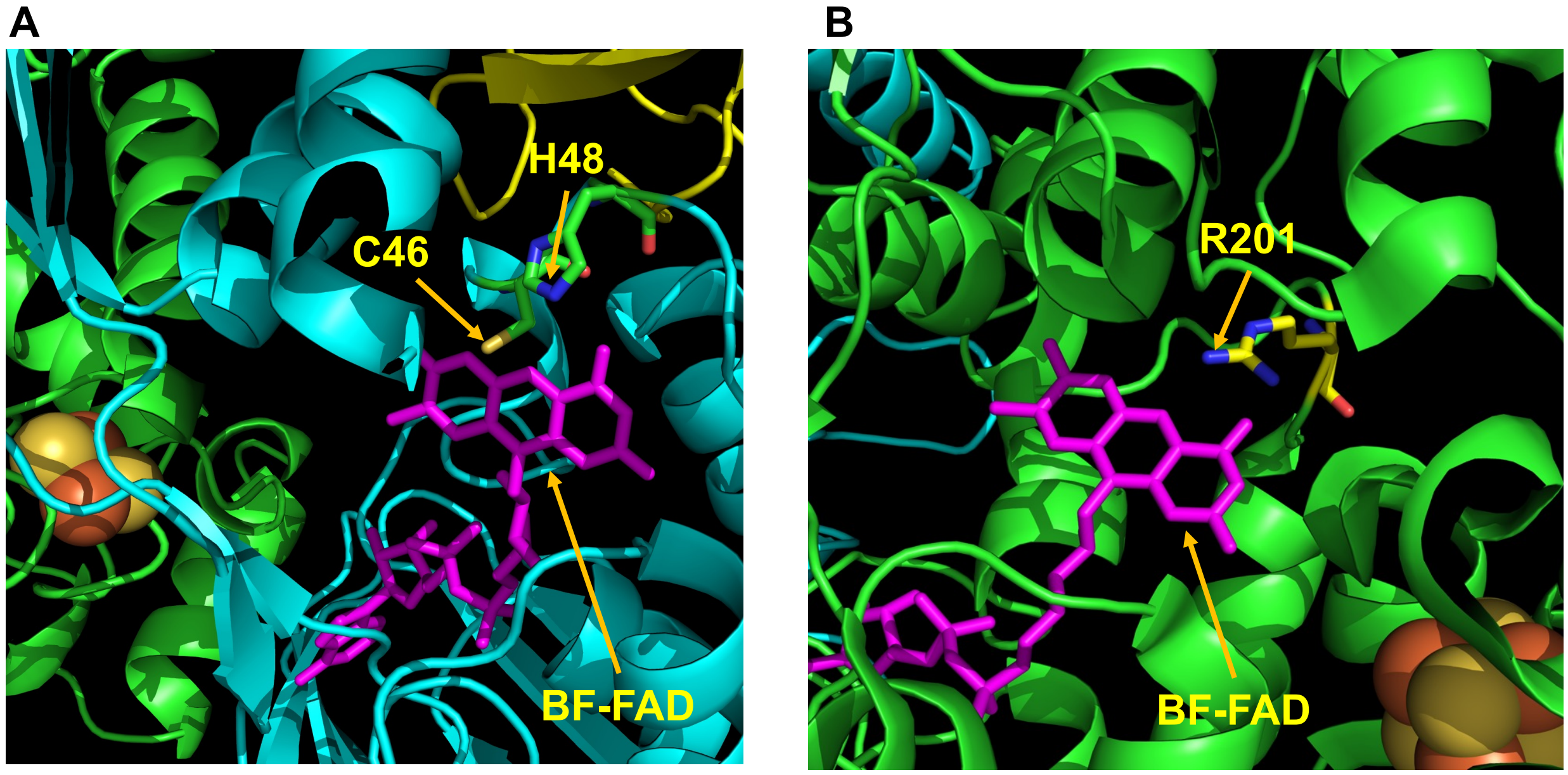

Figure S3. Comparison of the BF-FAD sites of FngABC and NfnSL from *P. furiosus*. A. Environment of the proposed BF-FAD in the FngB subunit. The positions of the cysteine (C46) and histidine (H48) residues adjacent to the FAD that are part of the conserved Gln-Cys-Ile-His motif are indicated. The S atom of C46 and the N atom of H48 are 4.6 Å and 2.9 Å away from the isoalloxazine ring system. The [2Fe-2S] cluster from FngA is shown by the yellow (S) and orange (Fe) space fill. **B.** Environment of the BF-FAD in the NfnL subunit (PDB id: 5JFC). The position of the conserved arginine (R201) is indicated where its terminal N atom is 2.9 Å away from the BF-FAD isoalloxazine ring system. The [4Fe-4S] cluster of NfnL is shown as yellow and orange space fill.

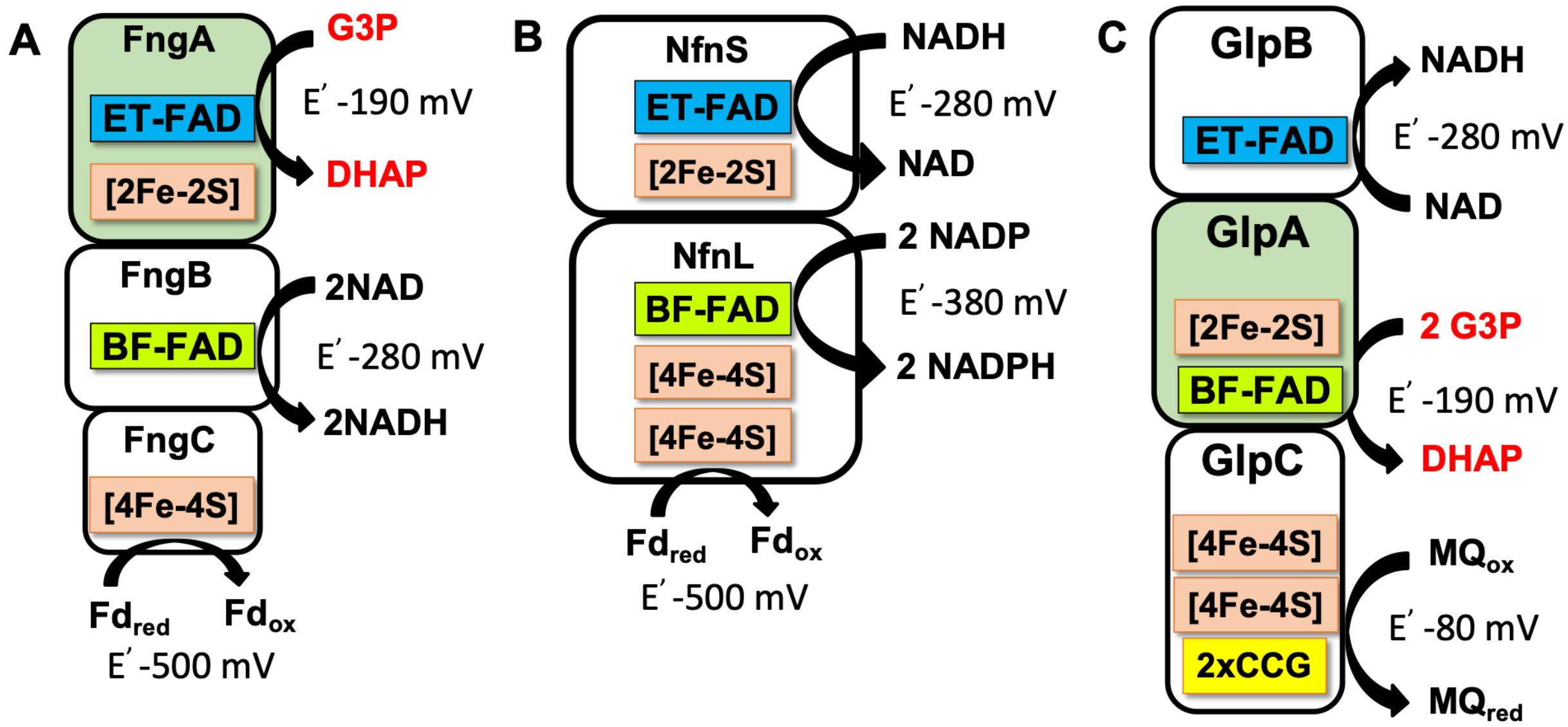

Figure S4. **Comparison of FngABC, NfnSL and *E. coli* GlpABC.** Cofactor contents and proposed electron bifurcation reactions are shown for A) *P. furiosus* FngABC (this work), B) *P. furiosus* NlnSL (ref 1), and C) predicted from the gene sequences of *E. coli* GlpABC (refs. 2 and 3). The effective potentials (E′) for each half reaction under physiological conditions are indicated. From sequence analyses only FngA and GlpA are homologous (indicated by green shading). The G3P to DHAP reaction in FngABC and GlpABC are highlighted in red.

References

1. Lubner, C. E.; Jennings, D. P.; Mulder, D. W.; Schut, G. J.; Zadvornyy, O. A.; Hoben, J. P.; Tokmina-Lukaszewska, M.; Berry, L.; Nguyen, D. M. N.; Lipscomb, G. L.; et al. Mechanistic insights into energy conservation by flavin-based electron bifurcation. *Nat Chem Biol* **2017**, *13* (6), 655–659. DOI: 10.1038/nchembio.2348

2. Cole, S. T.; Eiglmeier, K.; Ahmed, S.; Honore, N.; Elmes, L.; Anderson, W. F.; Weiner, J. H. Nucleotide sequence and gene-polypeptide relationships of the glpABC operon encoding the anaerobic sn-glycerol-3-phosphate dehydrogenase of *Escherichia coli* K-12. *J Bacteriol* **1988**, *170* (6), 2448–2456. DOI: 10.1128/jb.170.6.2448-2456.1988

3. Varga, M. E.; Weiner, J. H. Physiological role of GlpB of anaerobic glycerol-3-phosphate dehydrogenase of *Escherichia coli*. *Biochem Cell Biol* **1995**, *73* (3-4), 147–153. DOI: 10.1139/o95-018

Table S1 (supplied as a separate Excel file). The 5,252 homologs of the *P. furiosus* FngA subunit grouped by taxonomic class and predicted complex type. Only 4,273 of the homologs could be classified into possible complexes, the remainder were added to the A Only group. The colored cells correspond to the colors in Figure 4 where the values that exceed ~10% of total.

Table S2 (supplied as a separate Excel file). The UniProt metadata and genome context analyses data for the 5,252 homologs of the *P. furiosus* FngA subunit. Only 4,273 of the homologs have genome context analyses data and that data was used to annotate the phylogenetic tree in Figure 4. This data was also used to produce Table S1 and Table S3.

Table S3 (supplied as a separate Excel file). The 5,252 homologs of the *P. furiosus* FngA subunit grouped by an organism's unique UniProt Proteome ID and predicted complex type, The results were then back referenced to the proteins accession to give a row of predicted complex type values. These are duplicated for organisms with multiple homologs. Only 4,273 of the homologs could be classified into possible complexes, the remainder were added to the A Only group. This data was used to annotate the phylogenetic tree in Figure 4.

Table S4 (see page S7 of the Supplementary Information).

Table S5 (supplied as a separate Excel file). **Search terms input file for the genome context analyses**. These were used with regular expression patterns to search the genome context of the 4,273 of the *P. furiosus* FngA subunit homologs exported from the EFI-GNT genome neighborhood analysis tool. The Options sheets shows possible options for various fields in the file.

Table S6 (supplied as a separate Excel file). Matches output file for the genome context analyses with EFI-GNT dataset matches to the search terms file (see Table S5). This file is also an input file for the final step of the genome context analyses where Table S2 is the final output file. The file includes metadata to the matches such as accession numbers, sequence length, gene direction, gene position, match count and search term information.

Table S4. Primers used for construction the *P. furiosus* FngABC overproducing strain.

Primer name	Primer sequence
DN.131	GGATGCTGGAGATCAACAGG
DN.132	GGTGTTCCTCAAACATTTTCAAGTTATCCCCCTCCTCAACTC
DN.133	GATTATTGGGAGGTGGAGAAAAATGCATCACCACCATCACCACCACC ATCACGGTATTAAGGTTGCGATAATTGGAGC
DN.134	TAACATCTGCCTCAATGAATCCG
DN.135	ATTGAGGATCCATGCATCACCACCATCACCACCACCATCACGGTATT A AGGTTGCGATAATTGGAGCG
DN.136	ACTATGCGGCCGCTTAGGCTTCTCTGGTGGC
DN.137	GTCAAGATCCATCTCAACACC
DN.138	GGAAGCGAGATTGGTATAGGAG
DN.139	CAAAGCCTGGGTGAATACAC
DN.140	AGTGGTTAATGACAGGCTCG
DN.141	GTTTCACCCTGTTCTACAACG
